# How Positive and Negative Emotions Promote Ritualistic Consumption Through Different Mechanisms

**DOI:** 10.3389/fpsyg.2022.901572

**Published:** 2022-04-28

**Authors:** Wei Song, Taiyang Zhao, Ershuai Huang, Wei Liu

**Affiliations:** ^1^Yatai School of Business Management, Jilin University of Finance and Economics, Changchun, China; ^2^Department of Psychology, School of Philosophy and Sociology, Jilin University, Changchun, China; ^3^School of Business Administration Research Center for Energy Economics, Henan Polytechnic University, Jiaozuo, China; ^4^Business School, Qingdao University, Qingdao, China

**Keywords:** ritualistic consumption, positive emotion, negative emotion, sense of control, interesting experiences

## Abstract

Ritualistic consumption refers to integrating ritual elements into the process of product design and usage. By conducting three studies, we find that ritualistic consumption can offer new and interesting experiences and help consumers gain a sense of control. Both positive and negative emotions can promote ritualistic consumption tendencies. However, their underlying psychological mechanisms are different. Specifically, positive emotion can arouse consumers’ desire for interesting experience and thus promotes their preference for ritualistic consumption, while negative emotion can arouse consumers’ need for control and thus promote their preference for ritualistic consumption. Our research results offer a theoretical contribution and practical inspiration for emotional marketing.

## Introduction

Ritual is an important part of consumers’ daily lives. It occurs in celebratory, social or religious settings and has been used by marketers and created by individual consumers in the marketplace (Wang et al., 2021). There are many types of rituals, ranging from the macro level – comprising holidays that draw on shared cultural values – to the micro level – involving individuals, dyads or families engaging in idiosyncratic ritualistic behavior (Rook, 1985). Marketing researchers mainly concentrate on the micro level of rituals and define it as a type of behavior – used by marketers or created by consumers – that is comprised of several steps performed in a fixed sequence with formality, rigidity and repetition and imbued with a sense of meaning (Wang et al., 2021). Accordingly, we define ritualistic consumption as behavior that integrates ritual elements into the process of product design and usage.

More and more businesses and brands have regarded creating ritual experiences for consumers as one of their important strategies to promote products and maintain relations with customers (Malefyt and Morais, 2010; Liu et al., 2021). For example, Oreo cookie recommends the “twist-lick-dunk” eating ritual to their consumers – a typical consumption ritual created by marketers (Wang et al., 2021). Guinness beer recommends to consumers a specific series of pouring rituals, which is also regarded as a typical case of ritualistic marketing (Malefyt, 2015). In addition to the ritualistic marketing strategies of businesses, consumers also independently create some ritualistic behaviors in the process of consumption. For example, some consumers create specific rituals to enjoy food, such as breaking a chocolate bar in half and unwrapping and eating the two halves separately (Vohs et al., 2013). When tasting tea, some consumers are willing to follow certain ritualistic procedures, such as a specific tea-making process and symbolic tea-making posture (Hobson et al., 2018). In view of the above phenomenon, ritualistic consumption has become a new research field worthy of more attention for both practitioners and theorists.

In this study, we argue that ritualistic consumption is favored by businesses and consumers because it can better meet the deep-seated emotional needs of consumers in comparison with ordinary, non-ritualistic consumption. Therefore, the emotional arousal can promote the individual’s ritualistic consumption preferences. We will explore the following three questions through empirical studies: (1) compared with ordinary consumption, what psychological needs can ritualistic consumption better meet? (2) Will positive emotions and negative emotions affect individuals’ ritualistic consumption? (3) What are the differential psychological mechanisms underlying these effects respectively?

## Theoretical Framework

### The Role of Ritualistic Consumption

Literature in the field of psychology has found that rituals can help individuals resist the influence of negative events. For example, in the process of evolution, various religious and cultural rituals play an important role in human survival, which can help resist their sense of powerlessness in the face of natural disasters (Homans, 1941). Even in modern society, people are still keen to participate in various religious and cultural rituals, because participating in these rituals can help them alleviate negative emotions such as fear, anger and pain, as well as help people recover their mental health (Jacobs, 1989). Ai et al. (2005) found that after the September 11 terrorist attacks in the United States, the frequency of American people’s ritualistic behaviors increased significantly. Romanoff and Thompson (2006) found that in the dying ritual, the pain of both patients and their families can be relieved. We propose that the reason ritual can play the role of defending against negative events is that it improves an individual’s sense of control in two ways: First, the ritualistic behavior includes a series of repetitive actions, which can improve individuals’ sense of control (Lang et al., 2015). Second, rituals have rich symbolic meanings which include religious, divine or supernatural elements (Homans, 1941). Individuals can get a sense of power and control from these symbolic meanings (Kay et al., 2009). We argue that integrating ritual elements into the process of consumption can also play a similar role. Accordingly, we propose the following hypothesis:

**H1**: Consumers perceive more control when engaging in ritualistic consumption in comparison with ordinary consumption.

Individuals use rituals to resist negative events as well as in positive situations, such as birthdays, weddings and the commencement of a new year. Previous studies have found that participating in religious rituals can improve individual happiness, accompanied by the dopaminergic reward system (Alcorta and Sosis, 2005; Anastasi and Newberg, 2008). Vohs et al. (2013) found consumers will perceive a food or beverage as tastier and will enjoy and savor the experience of consumption when it is preceded by a ritual. The reason rituals bring pleasant experiences to people may be that they contain many interesting and attractive elements, which makes individuals more involved in their activities (van der Hart, 1983; Vohs et al., 2013). Wang et al. (2021) also found ritualistic consumption can increase consumers’ meaning in life, which brings a feeling of interest (Morgan and Farsides, 2009). Accordingly, we propose the following hypothesis:

**H2**: Consumers feel more interested when engaging in ritualistic consumption in comparison with ordinary consumption.

### Negative Emotion and Ritualistic Consumption

Negative emotions, such as anxiety, fear, sadness and depression, often come from individuals’ negative assessment of their own state and the external environment – particularly in situations where people may feel a lack of control (Keeton et al., 2008; Christensen et al., 2019; Brailovskaia and Margraf, 2021; Elemo et al., 2021). Previous studies have found that a sense of control is important for preserving individuals’ mental health (Manturuk, 2012; Precht et al., 2021). Improving one’s sense of control can reduce people’s negative emotions such as anxiety, stress and depression (O’Connor and Shimizu, 2002; Kim and Fusco, 2015; Brandão et al., 2020). According to compensatory control theory, when an individual’s sense of control is being threatened, he or she is motivated to restore control and devote themselves to improving their control through various actions (Kay et al., 2009; Landau et al., 2015). Therefore, we propose that negative emotions are often accompanied by a lack of control, which arouses an individual’s need for control and corresponding action to restore it. Though previous studies have found many methods that can help people restore personal control (Whitson and Galinsky, 2008; Hamerman and Johar, 2013; Faraji-Rad et al., 2017), as we elaborated above section, ritualistic consumption can also serve as an effective measure to restore a sense of control. Accordingly, we propose the following hypothesis:

**H3**: Negative emotion can promote consumers’ ritualistic consumption by arousing their need for control.

### Positive Emotion and Ritualistic Consumption

In addition to negative emotions, positive emotions may also promote people’s participation in various rituals. For example, when people are happy, they are more willing to participate in and organize various ritualistic activities (Boyer and Liénard, 2006); this can explain why people often hold rituals at festivals or other happy times in their lives. In some cases, achieving positive emotions is one of the important motivations for individuals to participate in rituals (Arnould and Price, 1993). This may be because positive emotions often mean that a person’s state and environment are comfortable and safe, which promote his or her tendency to explore and seek interesting new experiences. For example, previous studies have found that positive emotions can promote people’s involvement in new jobs and their willingness to meet new challenges (Diener et al., 2020). Students with positive emotions are more interested in various learning activities (Volet et al., 2019). Consumers with positive emotions are more willing to participate in various tourism and leisure activities because positive emotions can arouse people’s interest in new experiences (Mitas et al., 2012; Mitas and Bastiaansen, 2018). As we elaborated in the above section, ritual consumption contains many interesting elements (Vohs et al., 2013), which can meet the psychological needs of consumers who seek new experiences. Accordingly, we propose the following hypothesis:

**H4**: Positive emotion can promote consumers’ ritualistic consumption by arousing their desire for interesting experience.

## Study 1

The main objective of Study One was to verify H1 and H2 using an experimental method.

### Experimental Procedure

Study 1 adopted a single factor (consumption type: ritualistic vs. normal) between-subjects design. Study 1 was conducted in a laboratory in multiple batches, and 160 undergraduates (M_*age*_ = 20.26, SD_*age*_ = 1.38, 72 males and 88 females) from a Chinese university were recruited for a monetary payment. These participants were randomly assigned to the two experimental groups to receive the following experimental manipulation.

We used the method of Wang et al. (2021) to manipulate consumption type. Specifically, participants were told that this was a tasting activity for a new tea product, and they were asked to make and taste an actual pot of tea in the lab following instructions we provided. In the ritualistic consumption condition, participants were told that there are some tea-making and tasting rituals in Chinese culture, and this tea brand recommends consumers make and taste tea according to the corresponding ritual. The participants were asked to follow the following specific ritualistic steps to make and taste the tea: (1) pour hot water on the teapot for 15 s to raise the temperature of the teapot; (2) put the tea into the teapot; (3) pour in enough water to top off the teapot; (4) use the lid of the teapot to scrape the foam off the teapot mouth; (5) close your eyes to meditate for 3 min; (6) after 3 min, pour the brewed tea into the cup while gently and slowly shaking the tea in front of your face so that the aroma of tea can enter your nose; (7) drink the tea and feel the taste with the tip of your tongue. Conversely, in the normal consumption condition, the participants were simply instructed to make and taste the tea in any way they wished. Before tasting the eat, they were also asked to close eyes to meditate for 3 min, which used for balancing the possible interference of meditation to the result.

After the above procedure, we used the following three items, which referred to Vohs et al. (2013) in regard to measuring participants’ perceived interest: (1) This experience bored me (reverse-coded); (2) This experience was fun; (3) This experience was intrinsically interesting. The Likert 5-point scale was used (1 = totally disagree, 5 = totally agree), and the average score served as the indicator of perceived interest (α = 0.87). Perceived control was also measured using the following four items derived from Norton and Gino (2014): (1) To what extent did you feel out of control? (2) To what extent did you feel a sense of helplessness? (3) To what extent did you feel things were in check? (4) To what extent did you feel powerless? A 5-point scale was used (1 = not at all, 5 = very much), and the first, second and fourth items were reverse-coded. We averaged these items to create a composite measure of perceived control (α = 0.78). Finally, to conduct the manipulation check, participants were asked to use a 5-point scale (1 = not at all, 5 = very much) to rate the extent to which the experience invoked a sense of ritual.

### Results

#### Manipulation Check

The results of the manipulation check showed that the participants in the ritualistic consumption condition perceived a higher sense of ritual from the procedure (*M* = 4.21, *SD* = 0.67) than those in the normal consumption condition [*M* = 2.65, *SD* = 0.81; *t*(158) = 13.27, *p* = 0.000]. This result suggested that our manipulation of consumption type was successful.

#### Main Results

The results showed that the participants in the ritualistic consumption condition perceived the procedure as more interesting (*M* = 3.47, *SD* = 0.92) than those in the normal consumption condition [*M* = 2.40, *SD* = 0.81; *t*(158) = 7.76, *p* = 0.000]. This suggests that consumers perceive more interest when engaging in ritualistic consumption in comparison with ordinary, non-ritualistic consumption. Moreover, participants in the ritualistic consumption condition also reported a higher sense of control (*M* = 3.34, *SD* = 0.72) than those in the ordinary consumption condition – [*M* = 3.02, *SD* = 0.69; *t*(158) = 2.83, *p* = 0.005]. This suggests that consumers perceive a greater sense of control when engaging in ritualistic consumption in comparison with ordinary consumption. Thus H1 and H2 were supported.

## Study 2

Study Two provided an initial test of H3 and H4 by using a survey method.

### Sample and Measures

A total of 238 adult participants (139 females and 99 males; M_*age*_ = 29.63, SD_*age*_ = 7.09) were recruited from an online survey platform (“Credamo”) for certain monetary payment.

Our independent variable was positive and negative emotion. We first used the Positive and Negative Affect Schedule (PANAS; Watson et al., 1988) to measure participants’ current emotional state. The Cronbach’s alphas of positive and negative affect scales were 0.84 and 0.75. The average score of these two scales were used as the indicators of positive and negative emotion, respectively.

Then, we measured ritualistic consumption tendency, which served as our dependent variable. The participants were asked to read about an eating ritual for Oreo cookies. The brand recommends a “twist-lick-dunk” eating ritual to their consumers: Step 1 is twist – With each half of the Oreo in your fingertips, you begin to smoothly rotate your hands in opposite directions; Step 2 is lick – You lick the filling off; Step 3 is dunk – You dunk the cookie in milk and eat it (Wang et al., 2021). Then, the participants were asked to imagine they were preparing to eat an Oreo cookie and to rate the extent they wanted to follow the above recommended eating ritual on a 5-point scale (1 = not at all, 5 = very much).

Finally, we measured the need for control and the desire for interesting experiences, which served as our mediating variables. Need for control was measured by the following three items, as used by Gong and Zhang (2021): (1) I like to be in charge of everything in my life; (2) I like to make my own decisions and not be too influenced by others; (3) I don’t like feeling controlled by other people. The average score of the three items was used (α = 0.76). Desire for interesting experiences was measured by the following five items, as referenced by Ryakhovskaya et al. (2022): (1) I enjoy exploring new things; (2) I find it fascinating to have new experiences; (3) I enjoy learning about subjects that are unfamiliar to me; (4) I want to seek things that interest me; (5) Having interesting experiences is important to my life. The average score of the five items was used (α = 0.83). We also collected information about participants’ age, gender and education level as control variables.

### Results

We established regression models to test the main effects and mediating effects (see Table 1 for details). The results of the main effects are shown in Model 1. We found that both positive emotion (β = 2.66, *p* < 0.001) and negative emotion (β = 2.63, *p* < 0.001) had a significant positive impact on ritualistic consumption tendency. These results suggest that both positive emotion and negative emotion can improve consumers’ ritualistic consumption tendency.

**TABLE 1:** Regression tests of the main effects and mediating effects (*N* = 238).

Variable	Ritualistic consumption	Need for control	Desire for interesting experiences	Ritualistic consumption
	
	Model 1	Model 2	Model 3	Model 4
Age	−0.149*	–0.068	–0.003	−0.140*
Gender (female = 0)	−0.139*	–0.004	–0.044	−0.133*
Education level	0.023	–0.061	–0.047	0.036
Positive emotion	0.266***	0.023	0.319***	0.224***
Negative emotion	0.263***	0.154*	–0.006	0.246***
Need for control				0.119*
Desire for interesting experience				0.123*
R^2^	0.158	0.031	0.108	0.184
Adj. R^2^	0.140	0.010	0.088	0.159
F	8.724***	1.501	5.593***	7.417***

*p < 0.05 and ***p < 0.001.

Next, we used the regression analysis method proposed by Baron and Kenny (1986) to test the mediating role of a need for control and a desire for interesting experiences. Model 2 shows that negative emotion had a significant positive impact on need for control (β = 0.154, *p* < 0.05), while the effect of positive emotion was not significant (β = 0.023, p > 0.05). Model 3 shows that positive emotion had a significant positive impact on desire for interesting experiences(β = 0.319, *p* < 0.001), while the effect of negative emotion was not significant (β = −0.006, p > 0.05). Model 4 shows that both a need for control (β = 0.119, *p* < 0.05) and a desire for interesting experiences (β = 0.123, *p* < 0.05) had positive effects on ritualistic consumption tendency. The above results suggest that desire for interesting experiences mediated the positive effect of positive emotion on ritualistic consumption tendency, while the need for control mediated the positive effect of negative emotion on ritualistic consumption tendency. That is to say, positive and negative emotion improved consumers’ ritualistic consumption tendency via different underlying mechanisms. Specifically, positive emotion seemed to promote consumers’ ritualistic consumption by arousing their desire for interesting experiences, while negative emotion seemed to promote consumers’ ritualistic consumption by arousing their need for control. Thus H3 and H4 were supported.

## Study 3

The main objective of Study Three was to verify H1 and H2 using an experimental method.

### Experimental Procedure

Study 3 adopted a single factor (emotion: positive vs. negative vs. neutral) between-subjects design. We recruited 196 undergraduates (M_*age*_ = 21.49, SD_*age*_ = 2.02; 97 males and 99 females) from a Chinese university for a monetary payment and randomly assigned them to the three experimental groups to receive the following experimental manipulation.

Since the existing research methods mainly focus on the experimental manipulation of specific emotions, we choose two typical emotions to represent positive and negative emotion to complete the experimental manipulation. We chose happiness and sadness as examples of positive and negative emotions, respectively, and adopted autobiographical recall as the manipulation method, following Siedlecka and Denson (2019). We asked the participants in the positive (negative) emotion group to recall and write down experiences of when they felt happy (sad) in 100 words. The participants in the neutral emotion group were asked to recall and write down common events that they experienced yesterday. Before writing the task, we reminded participants that they would not be paid if they do not follow the instructions to write irrelevant content. After that, we used the same material and scales as in Study 2 to measure participants’ ritualistic consumption tendency, need for control and desire for interesting experiences.

### Results

#### Manipulation Check

After the experiment, we asked two doctoral students to rate – using a 5-point scale (1 = very negative, 5 = very positive) – the degree of positive or negative emotions reflected in the written materials for each participants. The average score was used for the manipulation check (*r* = 0.73). The result of the manipulation check showed that the scores in the positive emotion condition (*M* = 3.87, *SD* = 0.65) were significantly higher than those in the neutral emotion condition [*M* = 3.12, *SD* = 0.83; *t*(129) = 5.74, *p* = 0.000]. And the scores in the negative emotion condition (*M* = 2.15, *SD* = 0.51) were significantly lower than those in the neutral emotion condition, *t*(129) = 8.05, *p* = 0.000. These results suggested that our manipulation was successful.

#### Main Effects

The results showed that the ritualistic consumption tendency of participants in the positive emotion condition (*M* = 3.29, *SD* = 1.01) was significantly higher than for those in the neutral emotion condition [*M* = 2.80, *SD* = 1.13; *t*(129) = 2.63, *p* = 0.01]. Moreover, the ritualistic consumption tendency of participants in the negative emotion condition (*M* = 3.32, *SD* = 1.16) was also significantly higher than for those in the neutral emotion condition, *t*(129) = 2.60, *p* = 0.01. These results suggested that both induced positive and negative emotion can improve consumers’ ritualistic consumption tendencies.

#### The Mediating Effects

We used a bootstrap procedure to verify the mediating effects of need for control and desire for interesting experiences. This procedure computed a 95% confidence interval (CI) for the indirect and direct effects through 5,000 sampling. If a CI does not include 0, it indicates that the effect is significant (Preacher et al., 2007).

We first employed Model 5 in which positive emotion served as an independent variable (positive emotion condition = 1, neutral emotion condition = 0). As shown in Table 2, the results showed that the indirect effect of desire for interesting experiences (DI) between positive emotion (PE) and ritualistic consumption tendency (RC) was significant (95% CI: 0.046 to 0.336), while the indirect effect of need for control (NC) was not significant (95% CI: –0.132 to 0.061). These results suggest that desire for interesting experiences mediates the positive effect of positive emotion on ritualistic consumption tendencies, while the need for control does not. In Model 6 of Table 2, negative emotion served as an independent variable (negative emotion condition = 1, neutral emotion condition = 0). The results showed that the indirect effect of need for control between negative emotion (NE) and ritualistic consumption tendency was significant (95% CI: 0.021 to 0.263), while the indirect effect of desire for interesting experiences was not significant (95% CI: –0.079 to 0.143). These results suggest that the need for control mediates the positive effect of negative emotion on ritualistic consumption tendency, while desire for interesting experiences does not. The above results replicate the conclusions of Study 2 and support H3 and H4.

**TABLE 2:** Results of bootstrap mediating effects analysis.

	Path	Effect	Boot SE	LLCI	ULCI
Model 5: Effect of positive emotion	PE→NC→RC	–0.022	0.048	–0.132	0.061
	PE→DI→RC	0.166	0.073	0.046	0.336
	Direct effect: PE→RC	0.346	0.185	–0.021	0.712
Model 6: Effect of negative emotion	NE→NC→RC	0.124	0.063	0.021	0.263
	NE→DI→RC	0.025	0.055	–0.079	0.143
	Direct effect: NE→RC	0.371	0.198	–0.021	0.764

## Discussion

In the current research, we conducted three studies and verified the proposed hypotheses. In Study One, we first verified that compared with common consumption items, ritualistic consumption can bring more interest to consumers as well as help them gain a sense of control. In Study Two and Study Three, using survey and experimental methods, respectively, we found both positive and negative emotion can promote consumers’ ritualistic consumption tendencies. However, their underlying psychological mechanisms were different. Specially, positive emotion can arouse consumers’ tendencies to seek interesting experiences, thus promoting their preferences for ritualistic consumption, while negative emotion can arouse consumers’ needs for control, thus promoting their preferences for ritualistic consumption.

### Theoretical and Practical Implications

Nowadays, emotional marketing is an emerging concept in marketing. Researchers and practitioners in this area mainly study how to arouse emotions in people to induce them to buy particular products or service (Consoli, 2010). Although some studies in this area have found positive effects of emotional marketing, such as improving consumer satisfaction, affecting consumers’ purchasing decisions and brand perception (Khuong and Tram, 2015; Hindarsah, 2021), empirical research in this field is still scarce – especially for specific types of consumption. In this study, we chose ritualistic consumption as the research object to explore the role of emotions on individuals’ ritualistic consumption and the underlying psychological mechanisms, which can expand the research questions and theoretical basis in the field of emotional marketing. Furthermore, the previous literature on ritualistic consumption focused on exploring the effects of integrating ritual into the process of marketing and consumption (Vohs et al., 2013; Wang et al., 2021), but ignored exploring what factors can affect individuals’ ritualistic consumption. Our study employed positive and negative emotion as the affecting factors and revealed how and why they promoted consumers’ ritualistic consumption, which can – to some extent – compensate for the above-mentioned theoretical gap.

Our study can also provide inspiration for practitioners of emotional marketing. First, for businesses selling ritual-related products and services, they can adopt marketing strategies to arouse consumers’ positive and negative emotions to promote consumers’ ritualistic consumption. However, it is worth noting that when arousing consumers’ negative emotions, ritualistic products and services can provide a sense of control, while arousing consumers’ positive emotions, can bring an interest in new experiences. Second, when consumers are full of emotions – positive or negative – businesses selling ordinary products and services may want to create a sense of ritual for consumers in the process of customer communication and marketing. Third, for consumers, when they are in positive emotions, they can choose ritualistic consumption to strengthen their positive experience. When they are in negative emotions, they can choose ritual consumption to restore their sense of control.

### Research Limitations and Future Research Directions

In this study, we explored the role of positive emotions and negative emotions at a general level. However, both positive and negative emotions contain various kinds of specific emotions, such as sadness, anxiety, happiness and excitement. There may be subtle differences in the impact and potential mechanism of these specific emotions on ritualistic consumption. In future research, we will explore and compare the role of these specific emotions in more detail.

In the empirical studies, following the definition and research method of previous literature in marketing area (Wang et al., 2021), we mainly concentrate on the ritualistic consumption that occurs in the process of product use. However, there are many other forms of rituals in daily life that company with consumption, such as religious rituals, cultural rituals and festival rituals. In the future, we will try to expand our research on these forms of rituals.

## Data Availability Statement

The raw data supporting the conclusions of this article will be made available by the authors, without undue reservation.

## Ethics Statement

The studies involving human participants were reviewed and approved by the School of Philosophy and Sociology, Jilin University. Written informed consent for participation was not required for this study in accordance with the national legislation and the institutional requirements.

## Author Contributions

WS designed the study and conceived and drafted the manuscript. TZ provided writing instruction. EH collected, analyzed, and interpreted the data. WL instructed the project. All authors contributed to the article and approved the submitted version.

## Conflict of Interest

The authors declare that the research was conducted in the absence of any commercial or financial relationships that could be construed as a potential conflict of interest.

## Publisher’s Note

All claims expressed in this article are solely those of the authors and do not necessarily represent those of their affiliated organizations, or those of the publisher, the editors and the reviewers. Any product that may be evaluated in this article, or claim that may be made by its manufacturer, is not guaranteed or endorsed by the publisher.
